# Supplementary material for: The Systems Biology Research Tool: evolvable open-source software
Source: BMC Syst Biol. 2008 Jun 29;2:55. doi: 10.1186/1752-0509-2-55 (PMC2446383; doi:10.1186/1752-0509-2-55)
Supplement: Additional file 1 — SBRT Archive. An archive of the current version of the Systems Biology Research Tool. [file 1752-0509-2-55-S1.zip › sbrt-1.4.0/doc/developers_guide/api/sbrt/shell/mng/geom/CdHarManager.html]

CdHarManager


|  |  |  |
| --- | --- | --- |
| **Overview** \| **Package** \| **Class** \| **Use** \| **Tree** \| **Deprecated** \| **Index** \| **Help** | |  |
| PREV CLASS   NEXT CLASS | **FRAMES**    **NO FRAMES**     **All Classes** |  |
| SUMMARY: NESTED \| FIELD \| CONSTR \| METHOD | DETAIL: FIELD \| CONSTR \| METHOD |  |


---


## sbrt.shell.mng.geom Class CdHarManager

```
java.lang.Object
  sbrt.shell.mng.AbstractProcessManager
      sbrt.shell.mng.geom.CdHarManager
```

**All Implemented Interfaces:**: KernelProcessManager<java.lang.String,java.lang.String>, ProcessManager<java.lang.String,java.lang.String>

---

``` public final class CdHarManager extends AbstractProcessManager implements KernelProcessManager<java.lang.String,java.lang.String> ```

This class is used to manage coordinate direction hit-and-run algorithms.

**Author:**
:   This class was written and documented by Jeremiah Wright while in the
    Wagner lab.

---

| **Field Summary** | |
| --- | --- |
| `static java.lang.String` | `CONSTRAINT_TOLERANCE`             The keyword used to denote the constraint tolerance. |
| `static double` | `DEFAULT_CONSTRAINT_TOLERANCE`             The default constraint tolerance. |
| `static double` | `DEFAULT_MIN_CHORD_LENGTH`             The default constraint tolerance. |
| `static java.lang.String` | `INITIAL_POINT_FILE`             The keyword used to denote the initial point file. |
| `static java.lang.String` | `MAX_OBSTRUCTED_MOVES`             The keyword used to denote the maximum number of obstructed moves to allow. |
| `static java.lang.String` | `MIN_CHORD_LENGTH`             The keyword used to denote the minimum chord length. |
| `static UnmodifiableSet<java.lang.String>` | `optionalKeywords`             The set of keywords understood, but not required, by this process manager. |
| `static java.lang.String` | `PLANES_FILE`             The keyword used to denote the name of the file containing the collection of sets. |
| `static java.lang.String` | `POINTS_PER_POINT`             The keyword used to denote the number of points generated for each point returned. |
| `static UnmodifiableSet<java.lang.String>` | `requiredKeywords`             The set of keywords required by this process manager. |

**Fields inherited from class sbrt.shell.mng.AbstractProcessManager**

`ALGORITHM, COMPLETED, DATA_HEADERS, DEV_NULL, ELAPSED_TIME, FILE_FORMAT, INPUT_FILE, INPUT_FILE_FORMAT, INPUT_FILE_NAME_FILE, ITERATIONS, OUTPUT_FILE_FORMAT, OUTPUT_FILE_NAME, OUTPUT_FILE_NAME_FILE, PERCENT, PERCENT_COMPLETED, SEED, ZERO_CUTOFF`


**Constructor Summary**

`CdHarManager()`


| **Method Summary** | |
| --- | --- |
| `void` | `execute(java.io.PrintWriter out)`             Executes the process this process manager controls and writes the results to the provided print writer. |
| `static double` | `getConstraintTolerance(ManagerInput<java.lang.String,java.lang.String> input, java.io.PrintWriter out)`             Parses and returns the value for `CONSTRAINT_TOLERANCE` in the provided manager input. |
| `static java.util.Map<java.lang.String,java.lang.Double>` | `getInitialPoint(ManagerInput<java.lang.String,java.lang.String> input, java.io.PrintWriter out, java.util.Set<java.lang.String> variables)`             Opens the file with the name denoted by `INITIAL_POINT_FILE` and return the vector it contains. |
| `static java.util.Map<java.lang.String,java.lang.Double>` | `getInitialPoint(java.lang.String fileName, MapFormat<java.lang.String,java.lang.Double> format, java.util.Set<java.lang.String> variables)`             Opens the file with the provided name and return the vector it contains. |
| `static java.util.Map<java.lang.String,java.lang.Double>` | `getInitialPoint(java.lang.String fileName, java.io.PrintWriter out, java.util.Set<java.lang.String> variables)`             Opens the file with the provided name and return the vector it contains. |
| `static java.util.Map<java.lang.String,java.lang.Double>` | `getInitialPoint(java.lang.String fileName, java.util.Set<java.lang.String> variables)`             Opens the file with the provided name and return the vector it contains. |
| `static int` | `getMaxObstructedMoves(ManagerInput<java.lang.String,java.lang.String> input, java.io.PrintWriter out)`             Parses and returns the value for `MAX_OBSTRUCTED_MOVES` in the provided manager input. |
| `static double` | `getMinChordLength(ManagerInput<java.lang.String,java.lang.String> input, java.io.PrintWriter out)`             Parses and returns the value for `MIN_CHORD_LENGTH` in the provided manager input. |
| `UnmodifiableSet<java.lang.String>` | `getOptionalKeys()`             Returns the set of keywords understood, but not required, by this process manager. |
| `static ComoFile<java.lang.String,java.lang.Double>` | `getOutputFile(ManagerInput<java.lang.String,java.lang.String> input, java.io.PrintWriter out, java.util.Set<java.lang.String> headers)`             Returns an output file with the name provided in the manager input. |
| `static ComoFile<java.lang.String,java.lang.Double>` | `getOutputFile(java.lang.String fileName, java.io.PrintWriter out, java.util.Set<java.lang.String> headers)`             Returns an output file with the provided name. |
| `static ComoFile<java.lang.String,java.lang.Double>` | `getOutputFile(java.lang.String fileName, java.util.Set<java.lang.String> headers)`             Returns an output file with the provided name. |
| `static java.util.Set<ParallelHyperplanes<java.lang.String>>` | `getPlanes(ManagerInput<java.lang.String,java.lang.String> input, java.io.PrintWriter out)`             Returns the list of sets contained in the file with the name contained in the provided input. |
| `static java.util.Set<ParallelHyperplanes<java.lang.String>>` | `getPlanes(java.lang.String fileName)`             Returns the list of sets contained in the file with the provided name. |
| `static java.util.Set<ParallelHyperplanes<java.lang.String>>` | `getPlanes(java.lang.String fileName, java.io.PrintWriter out)`             Returns the list of sets contained in the file with the provided name. |
| `static int` | `getPointsPerPoint(ManagerInput<java.lang.String,java.lang.String> input, java.io.PrintWriter out)`             Parses and returns the value for `POINTS_PER_POINT` in the provided manager input. |
| `CdHarProcess<java.lang.String>` | `getProcess()`             Returns the process controlled by this process manager. |
| `UnmodifiableSet<java.lang.String>` | `getRequiredKeys()`             Returns the set of keywords required by this process manager. |
| `static ApplicationException` | `interpret(ExceededLimitException e)`             Returns an `ApplicationException` whose message describes the error that ocurred. |
| `static ApplicationException` | `interpret(UnboundedVariableException e)`             Returns an `ApplicationException` whose message describes the error that ocurred. |
| `void` | `setInput(ManagerInput<java.lang.String,java.lang.String> input, java.io.PrintWriter out)`             Uses the provided input to prepare this process manager for execution. |

**Methods inherited from class sbrt.shell.mng.AbstractProcessManager**

`check, closeFile, closeRm, execute, getFileFormat, getFileFormat, getFileNameSource, getIterations, getSeed, getTextFileFormat, getTextFileFormat, getZeroCutoff, getZeroCutoff, parseInt, parseLong, printProgress, printProgress, printProgress, runProcess, setInput`

**Methods inherited from class java.lang.Object**

`clone, equals, finalize, getClass, hashCode, notify, notifyAll, toString, wait, wait, wait`

**Methods inherited from interface sbrt.shell.mng.ProcessManager**

`check, execute, setInput`

**Field Detail**

### DEFAULT\_CONSTRAINT\_TOLERANCE

```
public static final double DEFAULT_CONSTRAINT_TOLERANCE
```

:   The default constraint tolerance.

    **See Also:**: Constant Field Values

---


### DEFAULT\_MIN\_CHORD\_LENGTH

```
public static final double DEFAULT_MIN_CHORD_LENGTH
```

:   The default constraint tolerance.

    **See Also:**: Constant Field Values

---


### PLANES\_FILE

```
public static final java.lang.String PLANES_FILE
```

:   The keyword used to denote the name of the file containing the collection
    of sets.

    **See Also:**: Constant Field Values

---


### CONSTRAINT\_TOLERANCE

```
public static final java.lang.String CONSTRAINT_TOLERANCE
```

:   The keyword used to denote the constraint tolerance.

    **See Also:**: Constant Field Values

---


### INITIAL\_POINT\_FILE

```
public static final java.lang.String INITIAL_POINT_FILE
```

:   The keyword used to denote the initial point file.

    **See Also:**: Constant Field Values

---


### MIN\_CHORD\_LENGTH

```
public static final java.lang.String MIN_CHORD_LENGTH
```

:   The keyword used to denote the minimum chord length.

    **See Also:**: Constant Field Values

---


### POINTS\_PER\_POINT

```
public static final java.lang.String POINTS_PER_POINT
```

:   The keyword used to denote the number of points generated for each point
    returned.

    **See Also:**: Constant Field Values

---


### MAX\_OBSTRUCTED\_MOVES

```
public static final java.lang.String MAX_OBSTRUCTED_MOVES
```

:   The keyword used to denote the maximum number of obstructed moves to
    allow.

    **See Also:**: Constant Field Values

---


### requiredKeywords

```
public static final UnmodifiableSet<java.lang.String> requiredKeywords
```

:   The set of keywords required by this process manager.

---


### optionalKeywords

```
public static final UnmodifiableSet<java.lang.String> optionalKeywords
```

:   The set of keywords understood, but not required, by this process
    manager.


**Constructor Detail**

### CdHarManager

```
public CdHarManager()
```


**Method Detail**

### interpret

```
public static ApplicationException interpret(UnboundedVariableException e)
```

:   Returns an `ApplicationException` whose message describes the error
    that ocurred.

    :   **Parameters:**: `e` - an unbounded variable exception. **Returns:**: an `ApplicationException` wrapping the provided exception.

---


### interpret

```
public static ApplicationException interpret(ExceededLimitException e)
```

:   Returns an `ApplicationException` whose message describes the error
    that ocurred.

    :   **Parameters:**: `e` - an exceeded limit exception. **Returns:**: an `ApplicationException` wrapping the provided exception.

---


### getInitialPoint

```
public static java.util.Map<java.lang.String,java.lang.Double> getInitialPoint(ManagerInput<java.lang.String,java.lang.String> input,
                                                                               java.io.PrintWriter out,
                                                                               java.util.Set<java.lang.String> variables)
```

:   Opens the file with the name denoted by `INITIAL_POINT_FILE` and
    return the vector it contains.

    :   **Parameters:**: `input` - the input containing a value for `INITIAL_POINT_FILE`.: `out` - the print writer to which informative messages will be written.: `variables` - the variables required to be present in the file. **Returns:**: the vector contained in the file with the provided name. **Throws:**: `FormatException` - if the file does not contain a required variable, contains a single variable multiple times, or contains any other formatting errors.

---


### getInitialPoint

```
public static java.util.Map<java.lang.String,java.lang.Double> getInitialPoint(java.lang.String fileName,
                                                                               java.io.PrintWriter out,
                                                                               java.util.Set<java.lang.String> variables)
```

:   Opens the file with the provided name and return the vector it contains.

    :   **Parameters:**: `fileName` - the name of the file containing the initial point, that is, vector.: `out` - the print writer to which informative messages will be written.: `variables` - the variables required to be present in the file. **Returns:**: the vector contained in the file with the provided name. **Throws:**: `FormatException` - if the file does not contain a required variable, contains a single variable multiple times, or contains any other formatting errors.

---


### getInitialPoint

```
public static java.util.Map<java.lang.String,java.lang.Double> getInitialPoint(java.lang.String fileName,
                                                                               java.util.Set<java.lang.String> variables)
```

:   Opens the file with the provided name and return the vector it contains.

    :   **Parameters:**: `fileName` - the name of the file containing the initial point, that is, vector.: `variables` - the variables required to be present in the file. **Returns:**: the vector contained in the file with the provided name. **Throws:**: `FormatException` - if the file does not contain a required variable, contains a single variable multiple times, or contains any other formatting errors.

---


### getInitialPoint

```
public static java.util.Map<java.lang.String,java.lang.Double> getInitialPoint(java.lang.String fileName,
                                                                               MapFormat<java.lang.String,java.lang.Double> format,
                                                                               java.util.Set<java.lang.String> variables)
```

:   Opens the file with the provided name and return the vector it contains.

    :   **Parameters:**: `fileName` - the name of the file containing the initial point, that is, vector.: `format` - the format object used to parse the file.: `variables` - the variables required to be present in the file. **Returns:**: the vector contained in the file with the provided name. **Throws:**: `FormatException` - if the file does not contain a required variable, contains a single variable multiple times, or contains any other formatting errors.

---


### getPointsPerPoint

```
public static int getPointsPerPoint(ManagerInput<java.lang.String,java.lang.String> input,
                                    java.io.PrintWriter out)
```

:   Parses and returns the value for `POINTS_PER_POINT` in the
    provided manager input.

    :   **Parameters:**: `input` - the input containing a value for `POINTS_PER_POINT`.: `out` - the print writer to which a status message will be printed. **Returns:**: the parsed value for `POINTS_PER_POINT` in the provided input.

---


### getMaxObstructedMoves

```
public static int getMaxObstructedMoves(ManagerInput<java.lang.String,java.lang.String> input,
                                        java.io.PrintWriter out)
```

:   Parses and returns the value for `MAX_OBSTRUCTED_MOVES` in the
    provided manager input.

    :   **Parameters:**: `input` - the input containing a value for `MAX_OBSTRUCTED_MOVES`.: `out` - the print writer to which a status message will be printed. **Returns:**: the parsed value for `MAX_OBSTRUCTED_MOVES` in the provided input.

---


### getMinChordLength

```
public static double getMinChordLength(ManagerInput<java.lang.String,java.lang.String> input,
                                       java.io.PrintWriter out)
```

:   Parses and returns the value for `MIN_CHORD_LENGTH` in the
    provided manager input.

    :   **Parameters:**: `input` - the input containing a value for `MIN_CHORD_LENGTH`.: `out` - the print writer to which a status message will be printed. **Returns:**: the parsed value for `MIN_CHORD_LENGTH` in the provided input.

---


### getConstraintTolerance

```
public static double getConstraintTolerance(ManagerInput<java.lang.String,java.lang.String> input,
                                            java.io.PrintWriter out)
```

:   Parses and returns the value for `CONSTRAINT_TOLERANCE` in the
    provided manager input.

    :   **Parameters:**: `input` - the input containing a value for `CONSTRAINT_TOLERANCE`.: `out` - the print writer to which a status message will be printed. **Returns:**: the parsed value for `CONSTRAINT_TOLERANCE` in the provided input.

---


### getPlanes

```
public static java.util.Set<ParallelHyperplanes<java.lang.String>> getPlanes(java.lang.String fileName,
                                                                             java.io.PrintWriter out)
```

:   Returns the list of sets contained in the file with the provided name.
    The sets must be parsable using `PipeSetFormat`;

    :   **Parameters:**: `fileName` - the name of the file containing a list of sets.: `out` - the print writer to which status messages will be printed. **Returns:**: the list of sets contained in the file with the provided name.

---


### getPlanes

```
public static java.util.Set<ParallelHyperplanes<java.lang.String>> getPlanes(java.lang.String fileName)
```

:   Returns the list of sets contained in the file with the provided name.
    The sets must be parsable using `PipeSetFormat`;

    :   **Parameters:**: `fileName` - the name of the file containing a list of sets. **Returns:**: the list of sets contained in the file with the provided name.

---


### getPlanes

```
public static java.util.Set<ParallelHyperplanes<java.lang.String>> getPlanes(ManagerInput<java.lang.String,java.lang.String> input,
                                                                             java.io.PrintWriter out)
```

:   Returns the list of sets contained in the file with the name contained in
    the provided input. The sets must be parsable using `PipeSetFormat`;

    :   **Parameters:**: `input` - the input containing the name of the file containing a list of sets.: `out` - the print writer to which status messages will be printed. **Returns:**: the list of sets contained in the file with the provided name.

---


### getOutputFile

```
public static ComoFile<java.lang.String,java.lang.Double> getOutputFile(ManagerInput<java.lang.String,java.lang.String> input,
                                                                        java.io.PrintWriter out,
                                                                        java.util.Set<java.lang.String> headers)
```

:   Returns an output file with the name provided in the manager input.

    :   **Parameters:**: `input` - the manager input containing the desired name of the output file.: `out` - the print writer to which a status message will be printed.: `headers` - the variables whose values will be written to the output file. **Returns:**: an output file with the provided name.

---


### getOutputFile

```
public static ComoFile<java.lang.String,java.lang.Double> getOutputFile(java.lang.String fileName,
                                                                        java.io.PrintWriter out,
                                                                        java.util.Set<java.lang.String> headers)
```

:   Returns an output file with the provided name.

    :   **Parameters:**: `fileName` - the desired name of the output file.: `out` - the print writer to which a status message will be printed.: `headers` - the variables whose values will be written to the output file. **Returns:**: an output file with the provided name.

---


### getOutputFile

```
public static ComoFile<java.lang.String,java.lang.Double> getOutputFile(java.lang.String fileName,
                                                                        java.util.Set<java.lang.String> headers)
```

:   Returns an output file with the provided name.

    :   **Parameters:**: `fileName` - the desired name of the output file.: `headers` - the data headers. **Returns:**: an output file with the provided name.

---


### getRequiredKeys

```
public UnmodifiableSet<java.lang.String> getRequiredKeys()
```

:   Returns the set of keywords required by this process manager.

    :   **Specified by:**: `getRequiredKeys` in interface `ProcessManager<java.lang.String,java.lang.String>`
    :   **Returns:**: the set of keywords required by this process manager.

---


### getOptionalKeys

```
public UnmodifiableSet<java.lang.String> getOptionalKeys()
```

:   Returns the set of keywords understood, but not required, by this process
    manager.

    :   **Specified by:**: `getOptionalKeys` in interface `ProcessManager<java.lang.String,java.lang.String>`
    :   **Returns:**: the set of keywords understood, but not required, by this process manager.

---


### getProcess

```
public CdHarProcess<java.lang.String> getProcess()
```

:   Returns the process controlled by this process manager.

    :   **Specified by:**: `getProcess` in interface `KernelProcessManager<java.lang.String,java.lang.String>`
    :   **Returns:**: the process controlled by this process manager.

---


### setInput

```
public void setInput(ManagerInput<java.lang.String,java.lang.String> input,
                     java.io.PrintWriter out)
```

:   Uses the provided input to prepare this process manager for execution. As
    the input is processed, informative messages are printed to the provided
    print writer.

    :   **Specified by:**: `setInput` in interface `ProcessManager<java.lang.String,java.lang.String>`
    :   **Parameters:**: `input` - all of the information required to prepare this process manager for execution.: `out` - the print writer to which all messages will be printed.

---


### execute

```
public void execute(java.io.PrintWriter out)
```

:   Executes the process this process manager controls and writes the results
    to the provided print writer.

    :   **Specified by:**: `execute` in interface `ProcessManager<java.lang.String,java.lang.String>`
    :   **Parameters:**: `out` - the print writer where the results will be printed. **Throws:**: `java.lang.IllegalStateException` - if no input was provided to this process manager.


---


|  |  |  |
| --- | --- | --- |
| **Overview** \| **Package** \| **Class** \| **Use** \| **Tree** \| **Deprecated** \| **Index** \| **Help** | |  |
| PREV CLASS   NEXT CLASS | **FRAMES**    **NO FRAMES**     **All Classes** |  |
| SUMMARY: NESTED \| FIELD \| CONSTR \| METHOD | DETAIL: FIELD \| CONSTR \| METHOD |  |


---
